# Multiple Forms of Glutamate Dehydrogenase in Animals: Structural Determinants and Physiological Implications

**DOI:** 10.3390/biology5040053

**Published:** 2016-12-14

**Authors:** Victoria Bunik, Artem Artiukhov, Vasily Aleshin, Garik Mkrtchyan

**Affiliations:** 1 A.N.Belozersky Institute of Physicochemical Biology, Lomonosov Moscow State University, Moscow 19991, Russia; 2 Faculty of Bioengineering and Bioinformatics, Lomonosov Moscow State University, Moscow 19991, Russia; whitelord32br@gmail.com (A.A.); Aleshin_vasily@mail.ru (V.A.); g.v.mkrtchyan@gmail.com (G.M.)

**Keywords:** acetylation, adenylated thiamine triphosphate, glutamate dehydrogenase isoenzymes, glutamate dehydrogenase isoforms, glutamate dehydrogenase alternative splicing, nucleotide-dependent regulation

## Abstract

Glutamate dehydrogenase (GDH) of animal cells is usually considered to be a mitochondrial enzyme. However, this enzyme has recently been reported to be also present in nucleus, endoplasmic reticulum and lysosomes. These extramitochondrial localizations are associated with moonlighting functions of GDH, which include acting as a serine protease or an ATP-dependent tubulin-binding protein. Here, we review the published data on kinetics and localization of multiple forms of animal GDH taking into account the splice variants, post-translational modifications and GDH isoenzymes, found in humans and apes. The kinetic properties of human GLUD1 and GLUD2 isoenzymes are shown to be similar to those published for GDH1 and GDH2 from bovine brain. Increased functional diversity and specific regulation of GDH isoforms due to alternative splicing and post-translational modifications are also considered. In particular, these structural differences may affect the well-known regulation of GDH by nucleotides which is related to recent identification of thiamine derivatives as novel GDH modulators. The thiamine-dependent regulation of GDH is in good agreement with the fact that the non-coenzyme forms of thiamine, i.e., thiamine triphosphate and its adenylated form are generated in response to amino acid and carbon starvation.

## 1. Introduction

**Figure 1:**
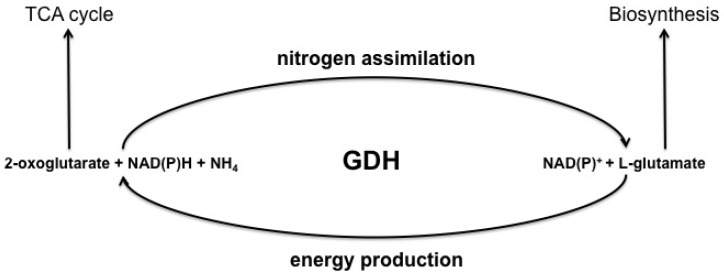
Schematic presentation of the reactions catalyzed by glutamate dehydrogenase and their general metabolic significance.

Glutamate dehydrogenase (EC 1.4.1.3; L-glutamate: NAD(P)^+^ oxidoreductase, deaminating) catalyzes oxidative deamination of glutamate in a bisubstrate NAD(P)^+^-dependent reaction, releasing 2-oxoglutarate, NH_3_ and NAD(P)H [1,2,3,4,5]. The enzyme also catalyzes the NAD(P)H-consuming backward reaction of the 2-oxoglutarate reductive amination to glutamate (Figure 1). In animals, both NAD^+^ and NADP^+^ may be electron acceptors [1,4,5,6].

The prevalent direction of the GDH reaction is determined by cell- and tissue-specific metabolic networks. In rat brain, the oxidative deamination of glutamate by GDH is favored [7,8,9,10,11,12,13,14]. However, neuronal GDH activity is two to five times lower than it is in astrocytes, where it must compete with highly expressed glutamine synthase that transforms glutamate to glutamine [15]. In liver, the glutamate dehydrogenase reaction is in equilibrium, whereas in pancreas and kidney, the enzyme catalyzes the oxidative deamination of glutamate to form 2-oxoglutarate [16,17,18].

There is a wealth of information on GDH within the various biological kingdoms. However, we focus our review on GDH from animal sources. Nevertheless, we shall also consider comparative aspects of general significance when discussing the metabolic roles and structure–function relationships of GDH. Characterization of such aspects in GDH from non-animal sources may be useful for understanding animal GDH. For instance, in contrast to animals, plants are thought to mostly employ the glutamate dehydrogenase trisubstrate (backward) reaction for nitrogen assimilation [19]. Indeed, this activity of plant GDH is elevated at increased concentrations of ammonia. The latter often occurs under stressful conditions, such as high temperatures, the absence of light, pollution and aging [20,21]. Under normal metabolic conditions, plants also use GDH in the bisubstrate (forward) reaction. Under these conditions, nitrogen assimilation is performed by the two enzymes catalyzing the glutamine: 2-oxoglutarate amidotransferase reaction using ferredoxin or NADH as electron donors (EC 1.4.7.1 and EC 1.4.1.14, correspondingly) [21]. Nevertheless, in some algae the GDH-dependent glutamate production is thought to be the dominant pathway of nitrogen assimilation under normal conditions [22].

## 2. Intracellular Localization of GDH in Animals

Glutamate dehydrogenase in eukaryotes was previously assumed to be localized in mitochondria only. Moreover, this enzyme was regarded as a mitochondria marker [23]. Active in the mitochondrial matrix, GDH may be inhibited by interaction with anionic phospholipids of the mitochondrial inner membrane, such as cardiolipin and phophatidylserine. These interactions mediate the binding of GDH to the inner mitochondrial membrane and probably regulate the interaction of this enzyme with a number of small molecules, including estrogens [24,25]. In the past, GDH activity in the nuclear fraction was considered as contamination of this fraction by mitochondria. Nevertheless, several studies [26,27,28,29,30] have pointed to a distinct pool of GDH associated with the nuclear fraction. The nuclear and mitochondrial enzymes exhibit different solubilization properties during purification and characteristic kinetic properties (see Section 5.1). In particular, the mitochondrial enzyme is solubilized in 0.25 M sucrose while the solubilization of the nuclear isoform requires the addition of 0.1 M potassium phosphate [26]. A recent study of chicken liver GDH [31] confirmed the nuclear localization of this enzyme [26,27,28,29] by uncovering the ability of GDH to act as a serine protease of histone H3 [31]. GDH was also found in the granular endoplasmic reticulum of rat liver [32]. Another form of GDH, which was localized in lysosomes and endosomes, has a tubulin-binding activity in the presence of ATP in vitro [33]. The colocalization of GDH with cytoskeletal elements (i.e., GFAP protein of intermediate filaments) has also been confirmed in human astrocytes [34].

The existence of membrane-bound GDH forms has been shown by various researchers [24,25,27,32]. For example, a membrane-bound isoform resistant to extraction by detergents such as digitonin, deoxycholate and Triton X-100, but solubilized with cationic detergents (hexadecyltrimethylammonium bromide), and to a lesser extent with 0.5 M NaCl or KCl, is present in rat brain [27]. Another form of GDH, resistant to extraction with Triton X-100, but partly extractable by 0.6 M NaCl, is present in rat liver endoplasmic reticulum [32]. GDH has also been identified in the plasmatic membrane of oligodendrocytes using immunocytochemical methods [35].

Several soluble forms of GDH that vary in thermostability and allosteric regulation are present in rat brain [27]. GDH is also detected in bovine thyroid gland cytosol, where it is thought to be involved in the synthesis of thyroid hormones [36].

## 3. Genomic, Transcriptomic and Proteomic Data on Multiple Forms of GDH in Animals

The multiple forms of GDH represent both different isoenzymes and isoforms: isoenzymes are the products of different genes, whereas isoforms are encoded by a single gene and arise through alternative splicing of mRNA or during or following translation. However, this distinction is often blurred in the literature. This section summarizes high-throughput data on structural variations in GDH, which could underlie the varied localization and properties of the enzyme considered above. It should be noted that the ability of different types of GDHs to perform different biological functions has long been known in plants, some bacteria as well as in higher and lower fungi [21,37,38]. For example, higher and lower plants possess GDH in chloroplasts [39,40], mitochondria and cytosol [21]. In *Neurospora crassa*, a hexameric NADP^+^-specific GDH is involved in anabolic processes. The enzyme has a low substrate specificity, aminating nine different 2-oxo acids in the biosynthesis of α-amino acids [41]. Another GDH in the same organism is NAD^+^-specific, involved in glutamate catabolism, and forms different oligomers than those of the NADP^+^-specific GDH [42]. On the other hand, both NAD^+^- and NADP^+^-specific bacterial GDHs (e.g., structures 3K92 from *Neurospora crassa* [43] and 3SBO from *Escherichia coli* [44], respectively) exist as hexamers and are homologs of animal GDHs.

### 3.1. Isoenzymes Encoded by Different Genes

Two distinct genes encode GDH isoenzymes in humans and higher primates [45,46,47]. One of these which encodes hGDH1, *GLUD1*, is located on chromosome 10, is expressed in all tissues, and contains introns. The other gene which encodes hGDH2, *GLUD2*, is intronless and is located on the X chromosome. This gene is expressed in neural tissue, kidneys and steroidogenic organs such as testes [48,49]. The ratio of the two isoenzymes differs. For example, in the liver only hGDH1 is present, whereas in Sertoli cells and cortical neurons only hGDH2 is present; astrocytes contain comparable amounts of both isoenzymes [48]. The subcellular distribution of the GDH isoenzymes also varies. Both isoenzymes are present in mitochondria, but only hGDH1 is associated with the nuclear membranes of astrocytes and oligodendrocytes [48]. Recombinant human GDH isoenzymes also differ in stability, including their heat resistance. Compared to hGDH1, hGDH2 is more labile upon dilution and at elevated temperatures [50]. Differences in the isoenzyme kinetics and regulatory properties [46,51,52] will be considered in Section 5.2.

Remarkably, hexamers of plant GDH are formed from α and β subunits, coded by different genes: *GDHA* (*GDH2*) encodes the α subunit and *GDHB* (*GDH1*) encodes the β subunit. Different proportions of the α and β subunits in the plant GDH hexamer are thought to contribute to multiple isoforms of GDH detected in plants [21]. The metabolic roles of the α and β subunits, as assessed by genetic means, were shown to differ. Elevated expression of the β subunit without affecting the level of α subunit results in increased catabolism of glutamate and a decrease in GDH-dependent nitrogen assimilation [21].

### 3.2. Other GDH Isoforms

**Figure 2:**
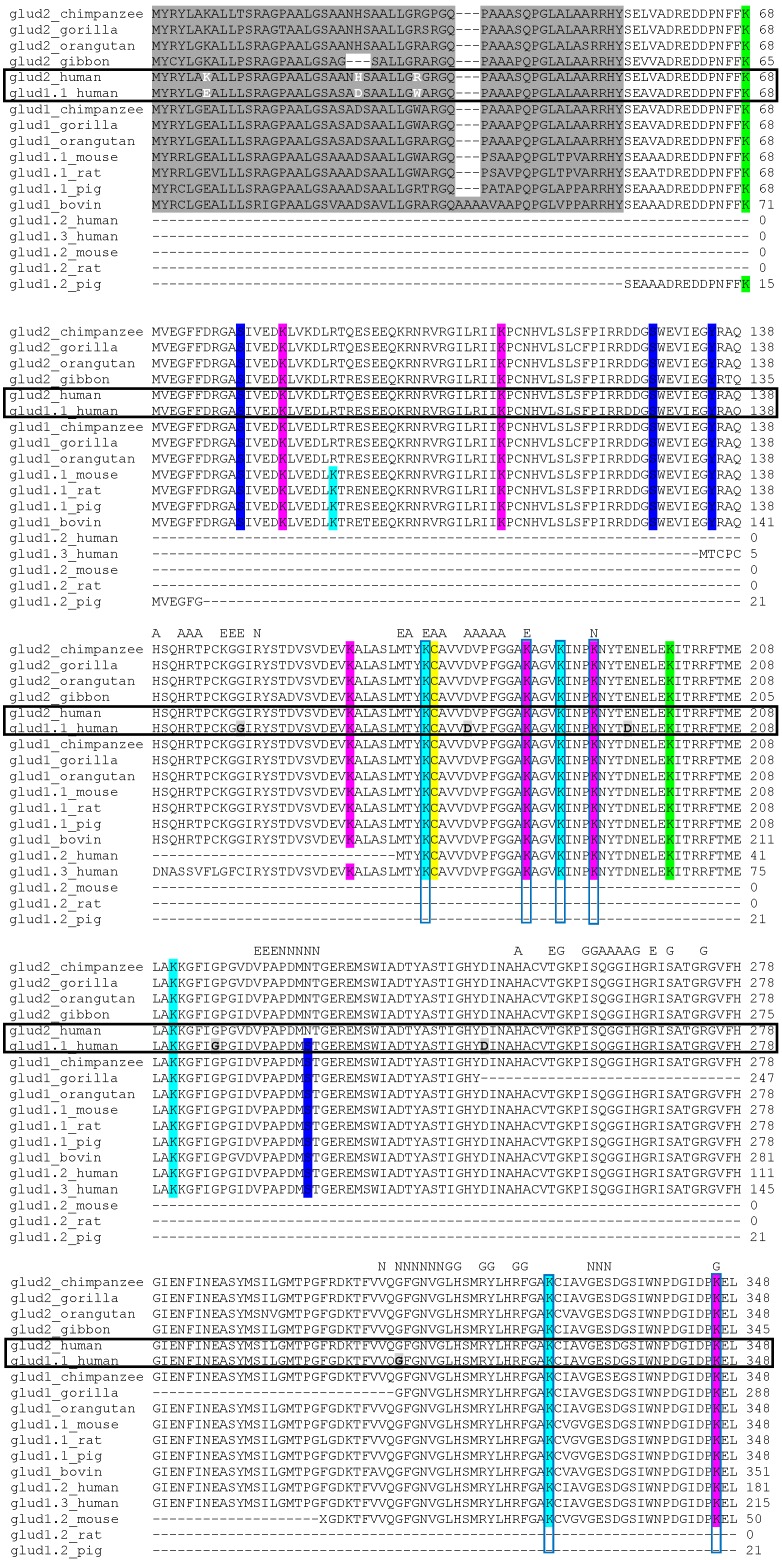

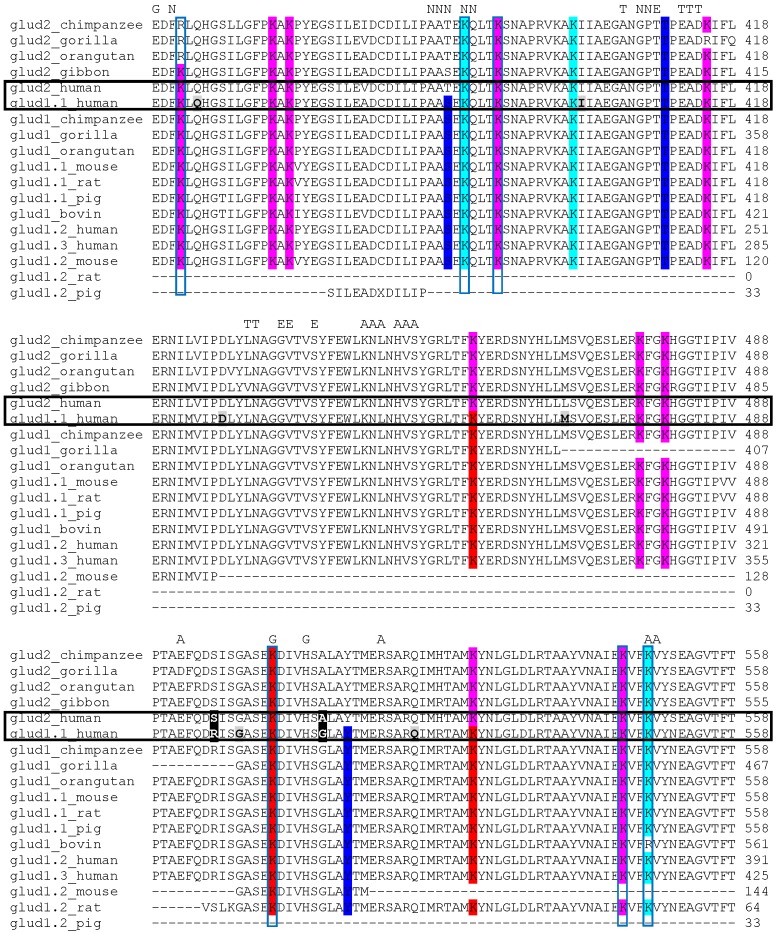
Multiple alignments of glutamate dehydrogenase isoenzymes and isoforms using ClustalW. Canonical sequences and/or their splice variants were extracted from UniProtKB protein knowledgebase (http://www.uniprot.org/). The sequences of the following proteins were used for the alignment: *Homo sapiens* (three splice variants of the *GLUD1* gene and the single product of *GLUD2* gene); apes, including *Pan troglodytes*, *Gorilla gorilla* and *Pongo abelii* (major product of *GLUD1* gene and the single product of *GLUD2* gene); *Hylobates lar* (only the product of *GLUD2* gene is included); *Rattus norvegicus*, *Mus musculus* and *Sus scrofa* (major and truncated products of *GLUD1* gene); *Bos taurus* (major product of *GLUD1* gene). Residue numbering includes the mitochondrial target peptide residues which are highlighted in dark grey. Electrostatic charge-affecting differences in the mitochondrial target peptide residues of human GDH isoenzymes are shown in white. The conservative ADP-ribosylated cysteine residue is highlighted in yellow. Serine, threonine and tyrosine residues subjected to phosphorylation are highlighted in dark blue. Lysine residues subjected to acetylation are highlighted in light blue, to succinylation—in green, to acetylation and succinylation—in pink, to acetylation, succinylation and malonylation—in red. Letters on the top mark the residues involved in the binding of GTP (“G”), NAD^+^ in the active site (“N”), ADP (“A”), glutamate (“E”) and forming the thiamine-binding motif (“T”). Lysine residues of established regulatory significance (discussed in the text) are vertically framed. Pairwise alignment of human glutamate dehydrogenase isoenzymes: hGDH2 (*GLUD2*) and hGDH1 (*GLUD1*) is horizontally framed. hGDH1 residues immediately before or within the triplets where the RNA splicing takes place, are marked in light grey and bold. Functional substitutions of hGDH1 residues resulting in the regulatory properties similar to hGDH2 are highlighted in black.

Apart from the isoenzymes encoded by several genes, alternative splicing increases the number of GDH isoforms transcribed from the same gene. In particular, four different RNA precursors [53] and four forms of GDH, varying in molecular mass and isoelectric point [54], are present in humans. These variants arise from two GDH genes [49]. Three protein-coding transcripts are known for the human *GLUD1* gene, while a single transcript encoded by the intronless *GLUD2* is responsible for the production of hGDH2. The major form of hGDH1 (glud1.1_human, Figure 2) consists of 13 exons. The intron-exon structure is conserved among different species. Figure 2 shows that the gorilla GDH1 sequence has two long deletions (Tyr247–Gly248 and Leu407–Gly408), compared to hGDH1 (Tyr247–Gly308 and Leu467–Gly499) and the GDH of other species. The deleted regions match the two different exons of hGDH1 (shown as the light grey residues in Figure 2). Hence, the GDH1 sequence determined in gorilla may be an alternatively spliced isoform of GDH1. Similarly, Figure 2 demonstrates that alternative splicing may result in the deletion of protein sequence between Pro128 and Gly129 in the glud1.2_mouse isoform. The three known alternatively spliced sequences of hGDH1 (glud1.1_human, glud1.2_human, glud1.3_human, Figure 2) are due to the differences in their first and second exons. While glud1.2_human may be a truncated form of glud1.1_human, the other variant, glud1.3_human, retains its specific first exon (Met1–Cys16), pointing to a difference from the canonical GDH sequence.

Remarkably, in the well-characterized products of alternative splicing in human (glud1.2_human and glud1.3_human) and gorilla (glud1_ gorilla), the deleted parts of the sequences comprise GDH residues involved in binding of glutamate and regulatory ligands ADP and GTP. In contrast, most of the residues for the catalytic NAD^+^ binding remain outside the deleted parts of the sequence (Figure 2). Given the absence of mitochondrial targeting sequences in glud1.2_human and glud1.3_human, such selectivity may underlie the moonlighting functions of these alternatively spliced isoforms of GDH1, such as a serine protease activity or tubulin-binding [33]. In contrast, the major GDH sequence has structural determinants for the ADP- and GTP-regulated glutamate-dependent reaction. The structural analysis of alternatively spliced GDH forms indicates that post-transcriptional modifications may be an important source of GDH isoforms, possessing different regulation and/or function.

GDH is also subject to a number of post-translational modifications, such as phosphorylation, acylation, ADP-ribosylation, proteolysis, and deamidation [54,55,56,57,58,59,60,61,62,63,64,65], which can further increase the structural and functional variety of GDH forms encoded by a single gene.

## 4. Overall GDH Structure and Structural Features of the Multiple Forms of GDH

### 4.1. Structural Studies of Mitochondrial GDH

**Figure 3:**
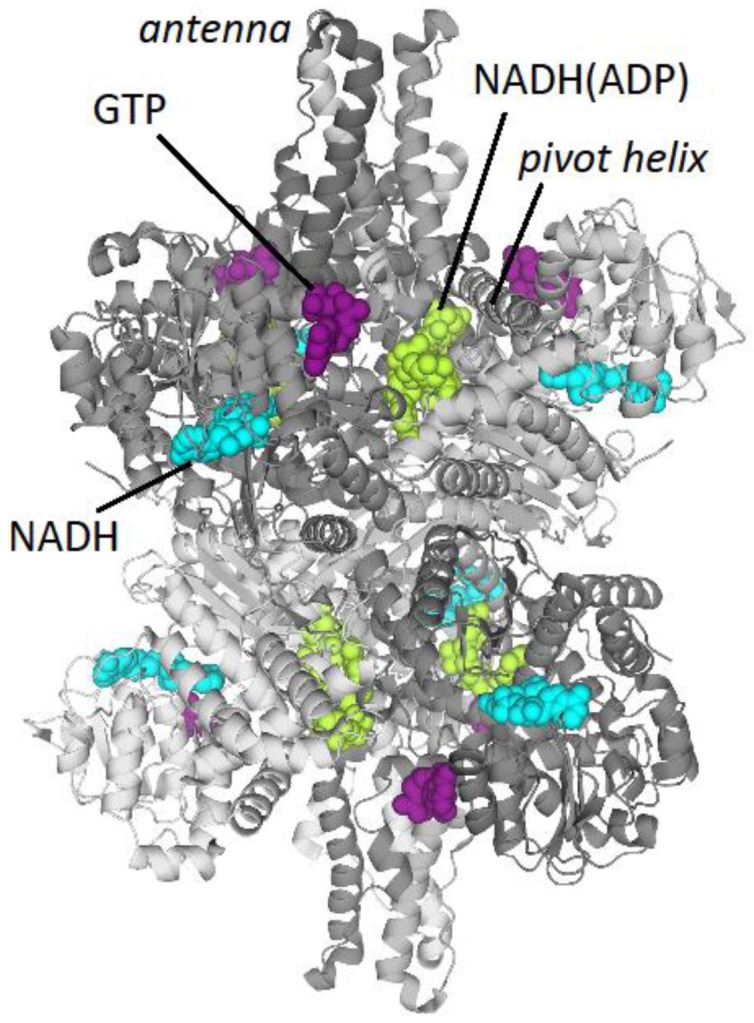
Structure of bovine glutamate dehydrogenase (PDB: 3JD4). Different subunits are shown in different shades of gray. Ligands are presented by space filling models: GTP in the allosteric inhibitory site (purple), NADH in the substrate-binding site (cyan) and NADH in the ADP allosteric site (green).

The structure of the mitochondria-localized mammalian GDH is well-studied. Mitochondrial GDH in animals exists as a homohexamer, with each subunit having a molecular mass of about 56 kDa. The enzyme consists of three main domains: the catalytic, NAD(P)^+^-binding and regulatory domains. The “core” of the enzyme is formed by the two trimeric N-terminal glutamate-binding domains, mainly arranged as β-strands (Figure 3). NAD^+^-binding domains are located nearby, with the rotating pivot helix regions (Figure 3) involved in catalysis [66]. Long α-helical regions, “antennae”, extend from the NAD^+^-binding domains. These trimeric “antennae” undergo conformational changes that open and close the enzyme active sites, and are unique to animal GDHs. The antennae also serve to facilitate the intersubunit communication that underlies the negative cooperativity and allosteric regulation of animal GDH [66]. However, absence of “antennae” in GDH from other species, such as eukaryotic GDH of *Neurospora* sp. or prokaryotic GDH from *Clostridia* sp. [67], does not equate to a lack of allosteric regulation in these GDHs. Indeed, the GDHs from other kingdoms may be allosterically regulated by other elements of the protein structure [68,69,70].

The open and closed conformations of bovine GDH have recently been resolved using cryoelectron microscopy [71]. Comparison of the structures in these two conformations clearly shows that the mobility of the catalytic NAD^+^-binding domains of GDH changes the ligand (NADH) position in the ADP allosteric site. The interdependent conformational mobility of the GDH catalytic and allosteric sites may underlie the ADP-dependent activation of GDH catalysis [71]. Information on the GDH regulation continues to emerge, including identification of novel sites for binding the green tea polyphenols and their analogs [72].

### 4.2. Sequence Alignment of Human GDH Isoenzymes and Mammalian GDH Isoforms

As seen from Figure 2, there are significant differences in leader peptides of the two hGDH isoenzymes which otherwise possess a high (95.7%) degree of sequence identity (Figure 2). Despite the same length of the mitochondrial targeting signals (53 amino acids), and the same length of the mature hGDH1 and hGDH2 (505 amino acids), the proportion of substitutions in the mitochondrial targeting signal (9/53) is much higher than that in the rest of the sequence (15/505). Moreover, the greater charge of the mitochondrial targeting signal in hGDH2, as compared to that of the hGDH1 isoenzyme, is due to Glu7Lys, Asp25His and Trp32Arg substitutions (Figure 2). As shown in the studies of mitochondrial transport, the charge of the mitochondrial target peptide is the most important for the mitochondrial localization of the transported protein. Thus, evolution of mitochondrial target peptide of hGDH2 seems to ensure its delivery to mitochondria [73,74].

As for differences in the mature proteins, several modifications have been found in mammals, which only apply to hGDH1. These modifications include phosphorylation of Ser227, which is substituted with Asn227 in GDH2 isoenzyme and therefore cannot be phosphorylated. Ser/Asn227 is localized in the NAD(P)H substrate binding site of GDH1 or GDH2, correspondingly (Figure 2). Such substitution obviously results in different phosphorylation of the GDH isoenzymes. Among other differences, highlighted in Figure 2, are substitutions in Ser384 in the NAD(P)H substrate binding site to Thr384 in most of GDH2s with the exception of the gibbon GDH2, where a Ser residue is present. Among apes (Figure 2), the chimpanzee, orangutan and gorilla GDH sequences are more closely related to human GDH sequences than to the gibbon sequences. Hence, the substitution of Ser384 to Thr in GDH2 sequences illustrates evolution of this enzyme in the hominoid taxonomic clade. Whether Ser384/Thr substitution is accompanied by altered regulation via phosphorylation is, however, unclear.

Other post-translational modifications of GDH1 isoenzymes include N6-malonylation of Lys457, Lys503 and Lys527 residues and phosphorylation of Tyr512. Although there are no data obtained for these modifications in GDH2 isoenzymes, based on the similarity of GDH1 and GDH2, the existence of these modifications in GDH2s cannot be excluded.

## 5. Kinetic Investigation of GDH and Its Isoforms

Most of kinetic studies on GDH were performed at the time when GDH was assumed to be localized to mitochondria and the existence of the enzyme isoforms was not taken into account. It should also be noted that the true dissociation constants of the enzyme-substrate complexes, *K_D_* values, are not necessarily equal to the Michaelis constants, *K_m_* values [75]. Nevertheless, kinetic parameters determined in such studies provide rough estimates for functional properties of different GDH preparations. In particular, such information is of practical significance when developing enzyme assays upon fractionation of different tissues or cell cultures. Table 1 depicts the substrate concentration ranges and maximal specific activities obtained with GDH purified from a variety of higher eukaryotic organisms. In view of a strong dependence of GDH kinetic parameters on the presence of multiple regulators, significant variations in these parameters determined in different studies are explicable. Consequently, the reaction conditions known to affect reaction rates of the GDH reaction are included in Table 1. Despite the variances, certain conclusions may be drawn from the data presented in Table 1. In particular, the *K_m_* values for glutamate and NH_4_^+^ vary much more than the *K_m_* values for 2-oxoglutarate. In NAD(P)^+^-dependent GDHs, the *K_m_* values for the reduced nucleotide NADH (10^−5^–10^−4^ M) are often an order of magnitude lower than the *K_m_* values for the oxidized nucleotide NAD^+^ (10^−4^–10^−3^ M), although the difference is not pronounced for the NADP^+^/NADPH couple in the same reactions. The feature is not obvious in either NAD^+^- or NADP^+^-specific GDHs.

### 5.1. Comparison of Kinetic and Regulatory Properties of Mitochondrial and Nuclear GDH

A comparative kinetic study of the mitochondrial and nuclear GDHs was performed with the enzymes isolated from rat liver [26]. The mitochondrial enzyme was solubilized in 0.25 M sucrose while the solubilization of the nuclear GDH in sucrose required the addition of 0.1 M potassium phosphate [26]. In contrast to mitochondrial GDH, the nuclear form was markedly activated by phosphate ions [26]. For example, a 12-fold activation of the nuclear GDH in the glutamate oxidation reaction was observed after addition of 0.2 M potassium phosphate at pH 9.0. In contrast, the mitochondrial enzyme showed a slight inhibition under the same conditions [26]. pH optima of the multiple forms in the forward and backward reactions did not significantly differ.

Table 2 shows that, compared to the mitochondrial GDH, nuclear GDH possesses a lower *K_m_* for glutamate, 2-oxoglutarate and NH_4_^+^, but a higher *K_m_* for NAD^+^. *K_m_* values for NADH are almost the same for the two enzyme forms. Similar properties of the mitochondrial and nuclear GDHs include inhibition by high concentrations of 2-oxoglutarate, NH_4_^+^ and NADH, and stimulation by ADP. However, high glutamate concentrations inhibit the nuclear enzyme only [26].

Recent data on both the nuclear and mitochondrial localization of the single human isoenzyme hGDH1 [31,96] suggest that the observed functional differences between the mitochondrial and nuclear GDHs may be due to post-transcriptional and/or post-translational modifications (see Section 3.2 and Section 7).

### 5.2. Kinetics and Regulation of GDH1 and GDH2

#### 5.2.1. Human Isoenzymes

Human GDH isoenzymes (hGDH1 and hGDH2) encoded by different genes (*GLUD1* and *GLUD2*), differ in their pH dependence. Compared to hGDH1, hGDH2 demonstrates a shift in pH optimum from 8.0 to 7.5 in the 2-oxoglutarate amination reaction [50,97]. The difference was suggested to be important in astrocytes, whose cytoplasm and mitochondrial matrix are acidified due to uptake of synaptic glutamate associated with counter-transport of OH^−^ ion [98,99,100].

Kinetic parameters for recombinant hGDH1 and hGDH2 [50,101] are presented in Table 3. The *K_m_* values for substrates exhibited by the two isoenzymes depend on their ADP saturation. At an intermediate concentration of ADP (0.1–0.25 mM), kinetic differences between the two isoenzymes are readily apparent (Table 3). The most obvious is a three-fold higher *K_m_* for glutamate, determined in the oxidative deamination direction, catalyzed by hGDH1, compared to that catalyzed by hGDH2. In contrast, the *K_m_* for 2-oxoglutarate determined in the direction of reductive amination, is higher for hGDH2 than for hGDH1.

The maximal velocities of glutamate oxidative deamination by the two isoenzymes are similar in the ADP-activated state, corresponding to 160 μmol/min per mg of purified enzymes at 1 mM ADP in the medium and 0.6–0.7 μmol/min per mg of protein in cell extracts with 0.5 mM ADP in the medium. The basal activity (i.e., in the absence of ADP) of hGDH2 is strongly dependent on protein concentration in the assay medium. Interestingly, a decrease in the assay temperature from 25 °C to 20 °C increased the basal activity of hGDH2 by an order of magnitude [50]. The inactivation of hGDH2 upon protein dilution, together with a high sensitivity of the hGDH2 activity to a small change in ambient temperature, suggests decreased stability of the oligomeric state of hGDH2 compared to that of hGDH1. Usually, a reversible dissociation of an oligomer is followed by irreversible denaturation of monomers, because the monomers are destabilized by the solvent exposure of their protein interfaces. The decreased stability of hGDH2 vs. hGDH1, which was observed in the reaction medium, could also contribute to a lower thermostability of hGDH2 vs. hGDH1 [46,50,102].

The two enzymes also differ regarding nucleotide-dependent regulation. Both isoenzymes are activated by ADP, but ADP activation is more pronounced for hGDH2 [51,101]. In particular, despite the fact that the SC50 value for ADP, i.e., the ADP concentration eliciting 50% of the maximum activation, is about three-fold higher for hGDH2 (58.7 μM) compared to hGDH1 (17.0 μM), the amplitude of the effect at saturating ADP concentrations (around 1 mM) is 10-fold higher for hGDH2 compared to hGDH1 [50]. The effect of another known allosteric activator, leucine, is also about 10-fold higher for hGDH2 compared to that for hGDH1, although SC50 values for leucine and their decrease upon ADP addition are similar in both hGDH isoenzymes [50].

GTP inhibits hGDH1, whereas hGDH2 is resistant to GTP (IC50 values for hGDH2 and hGDH1 are 78.5 μM and 0.31 μM, respectively) [50]. Resistance to GTP inhibition correlates with hGDH2 expression in tissues having high levels of mitochondrial GTP, such as brain and kidney [104]. Moreover, hGDH2 function is thought to be important in cells metabolizing large amounts of glutamate through the TCA cycle, such as astrocytes and Sertoli cells in brain and testes, respectively [101,105].

The two GDH isoenzymes are differentially inhibited by steroid hormones, polyamines and neuroleptics [52]. hGDH2 is about 20-fold more sensitive to one of the female sex hormones, 17β-estradiol (IC50 = 1.53 μM), than is hGDH1 (IC50 = 26.94 μM) [106]. It was observed that female patients with mutation Ser445Ala in the GTP-binding site of hGDH2 are somewhat protected from the early development of Parkinson disease (see Section 8.2) than are male patients. Compared to the wild-type hGDH2, the Ser445Ala variant of hGDH2 is more resistant to GTP inhibition. However, it also possesses an increased sensitivity to inhibition by estrogens, which might compensate for the GTP resistance, causing the protection in females [48]. Hormonal regulation of the human GDH isoenzymes is of great interest, but the structural basis of the GDH regulation by hormones of the steroidogenic pathway remains unresolved.

Spermidine, an endogenous polyamine compound, is also an endogenous GDH inhibitor, known to interact more potently with hGDH2 (IC50 = 2.8 mM) than with hGDH1 (IC50 = 6.3 mM) [107]. In addition to inhibition by estrogens, the spermidine effect could represent another mechanism which could “compensate” for the GTP resistance of hGDH2, compared to the GTP-sensitive hGDH1 isoenzyme [52]. Differential inhibition of hGDH isoenzymes by neuroleptics and its potential medical significance are discussed in Section 8.2.

To elucidate the molecular mechanisms that underlie these different regulatory patterns of the two isoenzymes, site-directed mutagenesis at positions containing different amino acid residues was performed. The results suggested that the low basal activity, heat-lability and increased sensitivity of hGDH2 to ADP, leucine and estrogens could be, at least partially, ascribed to the Arg443Ser substitution, whereas resistance to GTP inhibition has been attributed to the Gly456Ala substitution [50,108,109]. The mutated residues are shown in black in Figure 2, where their numbering, however, is different due to the contribution of the mitochondrial targeting signal.

Other amino acid substitutions studied thus far cannot explain all the functional differences between the two isoenzymes [110]. For instance, the Arg443Ser/Gly456Ala double mutation in hGDH1 did not result in the properties of wild-type hGDH2 [50]. This agrees with the notion that the regulatory properties are not always easily changed by substitutions in specific amino acid residues.

#### 5.2.2. GDH1 and GDH2 from Bovine Brain

Cho et al. [103] have described the kinetics of the two distinct GDH forms from bovine brain (bGDH1 and bGDH2). Regarding bGDH1 and bGDH2, the authors use the terms isoform and isoprotein as synonyms. Because no structural data are currently available to classify these GDHs as isoforms or isoenzymes according to the definitions given above, we shall refer to them as the enzyme forms. bGDH1 and bGDH2 were isolated from the total homogenate [103]. In view of the well-established multiple intracellular localizations of GDH (Section 2), this increases the potential for heterogeneity of the preparation compared to isolations from the mitochondrial fraction. Kinetic parameters of human GDH isoenzymes and identified forms of bovine GDHs are compared in Table 3. An analysis of the results presented in Table 3 indicates that the kinetic properties of bGDH1 and bGDH2, which were measured at a fixed ADP concentration (1 mM) resemble those of hGDH1 and hGDH2 isoenzymes at an intermediary ADP saturation (0.1–0.25 mM). In particular, a higher *K_m_* for glutamate and a lower *K_m_* for 2-oxoglutarate are inherent in both hGDH1 and bGDH1, compared to hGDH2 and bGDH2. The two human isoenzymes and bovine isoenzymes also show the same differences in *K_m_* for ammonium. Similar to hGDH2, allosteric activation by ADP was also more pronounced for bGDH2 under comparable conditions [103]. In addition, the *V_max_* for bGDH1 is higher compared to that for bGDH2 (202 and 124 μmol/min per mg of protein, respectively, in the presence of 1 mM ADP) which coincides with the high sensitivity of the hGDH2 isoenzyme to dilution, as discussed above.

Although a report that the relative thermostability of the human isoenzymes and bovine GDH forms was different (hGDH2 is less stable than hGDH1, and bGDH2 is more stable than is bGDH1), the thermostability was assayed under very different conditions, i.e., 100 mM sodium phosphate, pH 6.8, with 4 mg/mL bovine serum albumin, 47.5 °C for human GDH isoenzymes vs. 50 mM triethanolamine, pH 8.0, 42 °C for bovine GDH forms. The differences in relative thermostability could thus be due to phosphate ions, which are generally known as physiological regulators and, as shown above, significantly affect the nuclear form of GDH from rat liver in particular [26]. The thermostability is also known to depend on GDH binding of allosteric regulators [102] whose endogenous content could differ for GDHs isolated from bovine brain, compared to overexpressed human isoenzymes. In addition to the very different ionic conditions of the thermostability assays, one should take into account the different temperatures employed in the experiment, given the strong effect of only a 5 °C decrease in temperature on the stability of hGDH2 [50].

Overall, kinetic studies suggest that the two enzyme forms of GDH identified in bovine brain are functionally similar to the two human GDH isoenzymes.

## 6. Nucleotide-Dependent Regulation of Mammalian GDH and Its Relation to the GDH Regulation of Thiamine Compounds

In contrast to GDHs from bacteria, mammalian GDH is activated by ADP and inhibited by GTP [111].

### 6.1. ADP-Dependent Activation

ADP more readily binds to the “open” enzyme conformation than to the “closed” conformation, prevents the formation of the abortive GDH•NAD(P)H•glutamate and GDH•NAD(P)^+^•2-oxoglutarate complexes by decreasing the GDH affinity to the products of the reaction, and facilitates their release from the active site [112]. NAD(P)^+^/NAD(P)H non-catalytic binding to the ADP allosteric site also regulates the enzyme activity. The affinity of the allosteric ADP site toward NAD(H) is 10 times higher than that toward NADP(H) [113,114], and the reduced form binds more tightly than does the oxidized form [115].

### 6.2. GTP-Dependent Inhibition

The GTP inhibition site is available when the catalytic cleft is closed and GTP binds to the ”hinge” region of the NAD^+^-binding domain, increasing the energy required to open the catalytic cleft and release a product. Therefore, ADP and GTP bind in an antagonistic manner and GTP stabilizes the abortive complexes, inhibiting the reaction. GTP is unable to bind to the activator site for ADP. The antagonistic action of GTP and ADP on GDH is regarded as an enzyme “energy sensor” [112].

### 6.3. GDH Regulation by Thiamine Compounds

**Figure 4:**
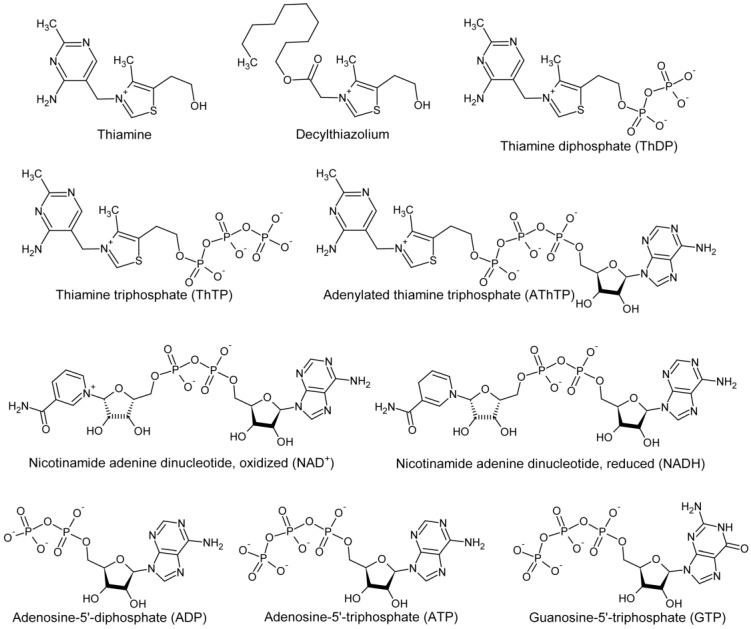
Structures of thiamine, its structural analog (decylthiazolium), natural derivatives of thiamine (ThDP, ThTP and AThTP), and the related nucleotides (NAD^+^, NADH, ADP, ATP and GTP).

GDH has recently been found to be among the most abundant enzymes in the fractions from bovine brain obtained by affinity chromatography using the thiazolium fragment of thiamine, (3-decyloxycarbonylmethyl-4-methyl-5-(2-hydroxyethyl)thiazolium, further referred to as decylthiazolium) (Figure 4), as a ligand [116]. Kinetic study of the regulatory effects of thiamine and its derivatives on GDH activity showed that thiamine diphosphate (ThDP), but not thiamine, inhibits glutamate dehydrogenase at non-saturating NADH concentrations [116]. Since the inhibitory effect decreases at NADH saturation, partial overlapping of the ThDP- and catalytic NADH-binding sites in GDH may be assumed. However, the inhibition by ThDP is significantly more pronounced in ADP-activated GDH, suggesting that ThDP competes with ADP at the allosteric activator site. It is thus probable that also in the absence of ADP, ThDP affects the substrate NADH site by binding to the allosteric site. ThDP binding to the ADP site, competitive with that of ADP, is consistent with certain structural similarities between the thiamine compounds and nucleotides, in particular, adenosine (Figure 4), and binding of thiamine to some adenosine sites [117]. In this regard, different regulation of the two GDH isoenzymes with nucleotides [50,101,110], discussed in Section 5, favors their different reactivity also to the thiamine compounds.

Although the inhibitory effect of ThDP is observed at relatively high ThDP concentrations (0.1–1 mM), the non-coenzyme thiamine derivatives, thiamine triphosphate (ThTP) and adenylated thiamine triphosphate (AThTP), act as GDH activators at micromolar concentrations [116]. We suggest that the non-coenzyme derivatives of thiamine are allosteric activators of GDH, whereas ThDP binding does not result in a conformation required for the activation effect, causing inhibition instead. Regulation of GDH activity by ThTP and AThTP is consistent with cellular synthesis of these non-coenzyme thiamine derivatives under metabolic stress, including amino acid and carbon starvation [118,119]. The biological significance of the thiamine regulation of GDH is supported in cellular experiments which demonstrated similar changes in GDH upon incubation of cells with thiamine and its antagonist, oxythiamine [120].

**Figure 5:**
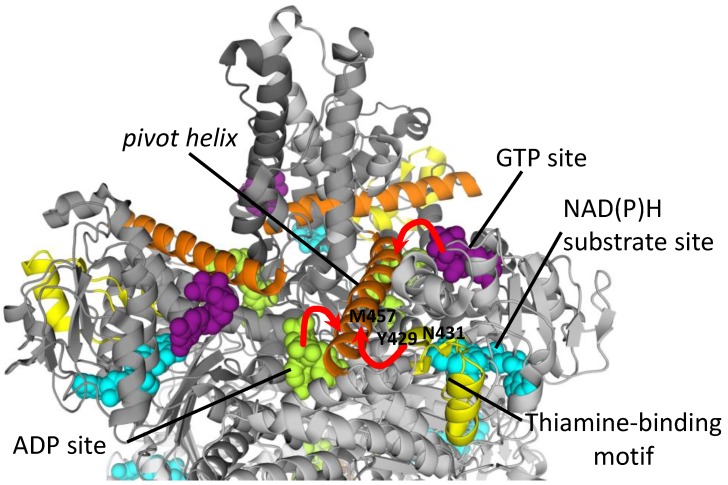
GDH regulatory sites. Different subunits of bovine glutamate dehydrogenase (PDB: 3JD4) are shown in different shades of gray. Space filling models depict GTP in the inhibitory center (purple), NADH in the substrate-binding center (cyan) and NADH in the ADP-activator center (green). A pivot helix is shown in orange, and the thiamine-binding motif described earlier [116],—in yellow. Positions of the residues of the thiamine-binding motif interacting with M457 of the pivot helix (Y429), and with the substrate binding site for NADH (N431) are indicated. The red arrows indicate interactions between the GDH allosteric sites, mediated by the pivot helix.

Due to the known interactions between the three nucleotide-binding sites of GDH (catalytic for NAD(P)H, activation for ADP and inhibition for GTP), kinetic determination of potential binding of the thiamine compounds to any of these GDH nucleotide binding sites does not seem feasible. As an alternative approach for localization of the binding site for thiamine compounds, the existence of the thiamine-binding sequence motifs in GDH was investigated [116]. After the thiamine-binding motifs were identified within the protein sequences, the 3D conformations of these motifs were assessed for similarity to their original 3D conformations in the thiamine-dependent protein templates [116]. One such motif with appropriate 3D conformation (Figure 5, shown in yellow) was found in close proximity to the glutamate- and NAD(P)H-binding sites (4.48 Å and 3.25 Å, respectively) [116]. The thiamine-binding motif is located at the edge of the typical βαβ-fold of NAD^+^-binding domain, proximal to several α-helices, including the pivot helix (Figure 5, orange). The N-terminal end of the pivot helix is involved in binding of GTP and ADP in one subunit, whereas its C-terminal end is close to the ADP-binding site of another subunit (Figure 5, indicated by red arrows). Therefore, localization of the thiamine-binding motif next to the C-terminus of the pivot helix suggests interaction of the thiamine site with both allosteric nucleotide-binding sites through the pivot helix (Figure 5, indicated by a red arrow). The interaction of the thiamine-binding motif (Figure 5, yellow) with the pivot helix (Figure 5, orange) is mediated by the hydrogen bond between the hydroxyl group of Tyr429 residue of the thiamine motif, and O-atom of Met514 of the pivot helix (in the mature GDH protein (3JD4) these residues are Tyr372 and Met457). Binding of the thiamine compounds by the motif may also regulate NAD(P)H binding in the substrate site, because another residue of the thiamine-binding motif, Asn431 (Asn374 in the GDH 3D structure 3JD4), is involved in the NAD(P)H binding at the substrate site [116]. The kinetic data shows that ThDP binding to GDH affects both the ADP-dependent activation of GDH and the substrate NADH binding. This supports the hypothesis that ThDP binds through the GDH thiamine-binding motif which interacts with both the ADP and substrate NADH sites through Tyr429 and Asn431, respectively.

Remarkably, the distance between the ADP-binding site and thiamine-binding motif of GDH may be covered by an extended conformation of AThTP (about 30 Å). The proximity of the two sites suggests a certain degree of conformational flexibility of the GDH structure involving the allosteric sites and thiamine-binding motif, allowing one to speculate that the ADP fragment of the GDH activator AThTP [116] is bound at the ADP activator site, while the thiamine fragment of AThTP is bound to the thiamine-binding motif. The terminal phosphate group(s) of the thiamine motif-bound ThTP might be able to interact with the diphosphate site of the ADP-binding center, providing for the observed ThTP activator effect, similar to that of AThTP [116]. However, the diphosphate group of ThDP appears to be too short to form a strong interaction with the diphosphate-binding residues of the ADP site. As a result, ThDP binding to GDH requires about a 100-fold higher concentration compared to ThTP and AThTP, and is unable to activate GDH.

## 7. Post-Translational Modifications of GDH and Their Biological Significance

### 7.1. NAD^+^-Dependent ADP-Ribosylation

**Figure 6:**
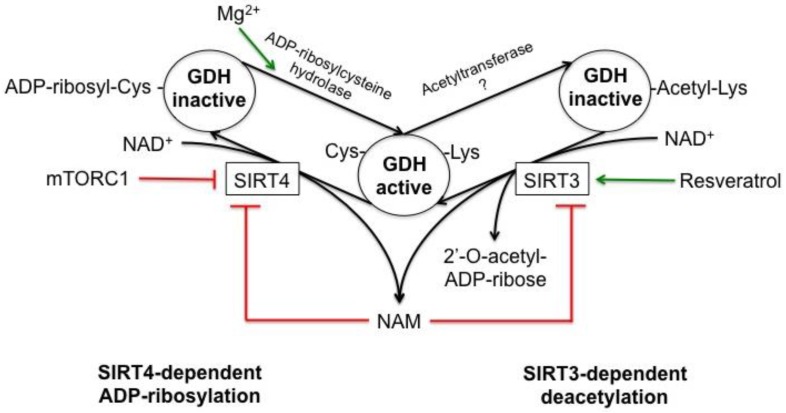
Schematic representation of GDH post-translational modifications regulated by sirtuins 3 and 4. ADP-ribosylation and acetylation are shown on the left and right, respectively. NAD^+^-dependent ADP-ribosylation of GDH is catalyzed by sirtuin 4 (SIRT4). Nicotinamide (NAM) is the second reaction product. As a result of the modification of a cysteine residue, GDH loses its catalytic activity. GDH deribosylation is catalyzed by ADP-ribosylcysteine hydrolase, which requires magnesium ion as a cofactor (indicated by a green arrow). The enzyme catalyzing acetyl-CoA-dependent GDH acetylation has not been identified, which is depicted as “Acetyltransferase ?”. GDH deacetylation is catalyzed by NAD^+^-dependent mitochondrial deacetylase, sirtuin 3 (SIRT3). NAD^+^ and acetylated GDH are the substrates for SIRT3 in the reaction releasing 2′-*O*-acetyl-ADP-ribose, NAM and deacylated GDH. The product of the sirtuin-dependent reactions, NAM, inhibits sirtuins. SIRT4 activity can be repressed via mTORC1 [122], and SIRT3 is up-regulated by resveratrol [123]. Red arrows indicate inhibition, whereas the green arrows represent activation.

GDH is the first mitochondrial protein shown to be ADP-ribosylated. Catalyzed by sirtuin 4 (SIRT4), this NAD^+^-dependent post-translational modification inactivates GDH. NAD^+^ is cleaved in the reaction, with the ADP-ribose moiety transferred to a cysteine residue of GDH and nicotinamide (NAM) released as the second reaction product (Figure 6). For mitochondrial liver GDH this modification has been shown to occur both in vitro and in vivo [121]. One conserved cysteine residue (marked yellow in Figure 2) is ADP-ribosylated in each GDH monomer. In human GDH Cys119 was identified as the ADP-ribosylated residue [58].

Isolated ADP-ribosylated GDH can be reactivated by Mg^2+^-dependent mitochondrial ADP-ribosylcysteine hydrolase (Figure 6). ADP-ribosylation of GDH may be part of a complex regulatory system controlling cellular nitrogen metabolism [121]. The physiological consequences of this regulation are discussed in Section 8.

### 7.2. Phosphorylation

Earlier studies showed the functional significance of GDH phosphorylation in several animal species. For example, GDH in *Otala lactea* snails is hyperphosphorylated during hibernation [124]. The phosphorylated form of the enzyme possesses a three-fold higher activity in the glutamate deamination reaction. The rate of the backward reaction is reduced by phosphorylation. Owing to this metabolic shift, ammonium ions, and subsequently, urea, accumulate in tissues of dormant snails. The ensuing increase in osmolarity prevents water loss in an arid environment. Moreover, the phosphorylated form of GDH has a reduced sensitivity to activation by ADP. As a result, at the low energy requirements of the dormant state, GDH-dependent NAD(P)H production does not respond to an increased ADP/ATP ratio as much as in the active state. In contrast, the sensitivity toward the inhibitor, GTP, is increased [124].

Phosphorylation of GDH from crayfish under hypoxic conditions leads to stimulation of the reductive amination of 2-oxoglutarate, reducing the tricarboxylic acid cycle activity and ATP production [125]. The phosphorylation also reduces the enzyme activation by ADP and increases inhibition by GTP.

GDH phosphorylation has also been found in mammals (gophers). In contrast to the snail GDH, phosphorylation of GDH in gophers is decreased during hibernation, with the dephosphorylated form of GDH shifting metabolism towards formation of 2-oxoglutarate and subsequent gluconeogenesis in liver [126].

Recent high-throughput proteomic studies identified multiple serine, threonine and tyrosine phosphorylation sites in mammalian GDHs [57,60,61,63,65]. However, the functional significance of specific GDH phosphorylation reactions remains unknown.

Remarkably, reversible inactivation of GDH by phosphorylation has long been known for the NAD^+^-specific enzyme from yeasts *Candida utilis* [127,128] and *Saccharomyces cerevisiae* [92,129]. In these species, phosphorylation of GDH, catalyzed by both the cAMP-dependent and cAMP-independent protein kinases, inactivates the enzyme. Reactivation via dephosphorylation is catalyzed by protein phosphatases. In contrast, phosphorylation of NADP^+^-specific GDH in the lower fungi *Benjaminiella poitrasii* was shown to activate the enzyme [130,131]. Differences in the GDH phosphorylation level were shown under nitrogen starvation (NAD^+^-specific GDH in *C. utilis* and *S. cerevisiae*) or in yeast and mycelium states (NADP^+^-specific GDH in *B. poitrasii)*. The findings suggested that phosphorylation of fungal GDH affects the ratio between the different GDH activities, which is important for metabolic regulation [92,131].

### 7.3. Lysine and Cysteine Acylation

Like many other metabolic enzymes, GDH has been shown to be acetylated [132]. Functional changes in GDH due to acetylation require further investigation. In this regard, earlier studies on chemical acetylation of GDH lysine residues in vitro are of interest. It has been shown [133] that GDH loses 80% of its catalytic activity after in vitro acetylation of one amino group per subunit, and the acetylation significantly weakens the interaction between the enzyme subunits. The resulting dissociation of the enzyme hexamer has been shown to cause altered allosteric regulation: affinity of the acetylated enzyme to zinc ions and GTP (both are GDH inhibitors) decreases, whereas that to ADP (activator) increases. Examination of putative acetylation sites (Figure 2) within the currently known 3D-structure of GDH (Figure 3) suggests that the consequences of the acetylation of a single amino group in GDH studied by Colman and Frieden [133], may occur upon acetylation of Lys155. This residue forms a hydrogen bond with Gly156 of the neighboring subunit. Breakdown of the hydrogen bond after acetylation of lysine residues may be responsible for the observed perturbation in subunit interactions causing altered allostery observed after acetylation of a single site [133].

At least 11 lysine residues of the GDH monomer were found to be acetylated in vivo [134,135]. Acetylation of Lys477 and Lys480, which are located in the antennae domain, may also affect the intersubunit interactions and allosteric behavior of GDH [136,137]. The level of GDH acetylation is regulated by an as yet unidentified mitochondrial acetyltransferases and a NAD^+^-dependent mitochondrial deacetylase, sirtuin 3 (SIRT3) (Figure 6). Incubation of GDH with recombinant SIRT3 in the presence of NAD^+^ results in enzyme deacetylation, while addition of NAM completely blocks the reaction [138]. Owing to this, the level of GDH acetylation can be controlled via modulating SIRT3 function (Figure 6), e.g., by the SIRT3 inhibitor NAM [139] and its activator resveratrol [123]. GDH in mitochondrial preparations obtained from a SIRT3-knockout rat was shown to be hyperacetylated [135]. Thus, multiple lines of evidence indicate that SIRT3 controls the level of GDH acetylation in vivo. The physiological consequences of the changes in the level of GDH acetylation are discussed in Section 8.

In addition to acetylation, GDH may be involved in other acylation reactions, such as succinylation, malonylation and fatty acid acylation. Many of the acylated residues of GDH may be either acetylated or succinylated, but some of the identified succinylation sites are distinct from the known sites of acetylation (Figure 2) [59,64]. Mitochondrial succinyltransferases have not been identified yet. However, desuccinylation is known to be catalyzed by NAD^+^-dependent mitochondrial desuccinylase, sirtuin 5 (SIRT5), which also possesses other deacylation activities [64,140,141].

Three lysine residues of bovine GDH were found to be malonylated in the commercial enzyme preparations (Figure 2) [64]. The malonylation may be catalyzed by yet unidentified mitochondrial enzyme(s) (malonyltransferase(s)), whereas demalonylation is catalyzed by SIRT5. A functional role for GDH malonylation or succinylation is unknown.

Finally, GDH may undergo acylation by long-chain acyl-CoAs at cysteine residues. In particular, GDH was shown to be acylated by myristoyl-CoA [142] and palmitoyl-CoA [143], with both modifications leading to enzyme inactivation. Acylations with fatty acids may occur either spontaneously or enzymatically via palmitoyl acyltransferases [144]. Removal of the long-chain acyl group is catalyzed by palmitoyl acyl thioesterases. These modifications are assumed to regulate targeting of proteins to certain cellular microdomains [143,144].

Functional consequences of acylation may include changes in GDH reactivity to allosteric regulators. For instance, different affinity to the allosteric regulators, including thiamine compounds, and varied amplitude of their effects on the activity of GDHs from different organisms or tissues may be due to organism- or tissue-specific modifications of lysine residues near the allosteric ligand-binding sites. In particular, in the major splice variants of hGDH1, Lys171, Lys545 and Lys548 are near the ADP-binding site, and Lys390, Lys415 and Lys503 are near the GTP-binding site. Lys161 and Lys183 in the same isoenzyme are close to the active site, and Lys187, Lys191 and Lys352 are in the vicinity of the NAD(P)(H) substrate-binding site of GDH (Figure 2).

Comparison of the modified lysine residues in GDH isoenzymes and isoforms (Figure 2) allows one to make the following conclusions. The product of the *GLUD2* gene has some lysine residues substituted by arginine residues when compared to the product of the *GLUD1* gene. Thus, these isoenzymes should differ in acetylation/succinylation patterns. The same applies to the splice variants of GDH, which possess only a subset of potentially modifiable lysine residues.

### 7.4. Oxidation and Nitration

Novel post-translational GDH modifications, oxidation and nitration, were also shown to significantly affect the GDH activity [145]. Both oxidation and nitration of GDH can be detected in the pathogenesis of numerous diseases [146,147,148,149,150]. The studies of these modifications demonstrated that nitrating agents inactivate bovine GDH. Depending on the agent used, different tyrosine residues were nitrated with different consequences for the GDH activity. For example, NO_2_^−^ promoted GDH inactivation via carbonylation, but increasing NO_2_^−^ concentrations restored GDH activity concomitant with decreased GDH carbonylation and increased nitration of Tyr262 and Tyr474 residues. The complex and reversible response of GDH to NO_2_^−^ may be of physiological significance regarding NO_2_^−^-mediated carbonylation and nitration. For instance, specific carbonylation of Na^+^/K^+^-ATPase was shown to be involved in signal transduction [151]. Another nitrating agent, 3-morpholinosydnonimine (SIN-1), also caused both GDH carbonylation and tyrosine nitration. However, in this case Tyr401 and Tyr493 residues were nitrated with increasing SIN-1 concentration, which caused no restoration of the GDH activity, but further decreased the activity due to increase in GDH carbonylation. Thus, GDH carbonylation which causes a decrease in enzyme activity is affected by nitration of specific tyrosine residues of GDH.

Earlier studies on in vitro tyrosine nitration of bovine GDH with tetranitromethane showed that nitration of one tyrosine residue per subunit (Tyr412), which later was correctly identified as Tyr464 (Figure 2), does not affect the activity of the enzyme but decreases its response to inhibition by GTP [152,153,154]. In the resolved bovine GDH structure, Tyr464 is located in the middle of the antennae region, which suggests that the nitration of this residue may abrogate conformational changes of the antennae, thus perturbing the allosteric regulation of GDH [66].

## 8. Medical Significance of GDH

### 8.1. GDH of Peripheral Tissues

In kidneys, as well as in many other organs, GDH helps to maintain the concentration of NH_4_^+^ ion [2,155,156]. The enzyme was also shown to be involved in the mechanism of insulin secretion in pancreatic β-cells [17]. It was shown that *GLUD1* mutations decrease the enzyme sensitivity to its inhibitor GTP, contributing to hyperinsulinism/hyperammonemia (HI/HA) syndrome, which results in decreased glucose and elevated NH_4_^+^ levels in blood [66,112,157,158,159]. The insulin release is linked to dysregulated production of 2-oxoglutarate by pancreatic β-cells with mutated GDH [158,160].

Remarkably, hyperinsulinism and hypoglycemia are also caused by mutations in the short-chain 3-hydroxyacyl-CoA dehydrogenase (SCHAD), which is involved in the oxidation of fatty acids [161,162]. Recently, SCHAD was shown to bind GDH from pancreatic islets and inhibit its activity [163]. Mutated SCHAD, however, is incapable of binding to GDH. The resulting activation of pancreatic GDH causes inappropriate insulin release [163], similar to that observed upon the *GLUD1* mutations causing impairment of the GDH inhibition by GTP [2,155,156].

Studies of ADP-ribosylation indicated a role of this modification in mediating the GDH impact on insulin production as well. For example, SIRT4-dependent ADP-ribosylation inactivates GDH in pancreatic β-cells, limiting the insulin release [164]. The inactivation of GDH may be reversed under a low level of glucose, resulting in elevation of the pancreatic GDH activity and insulin level in blood. Reduced ADP-ribosylation of GDH was found under caloric restriction, associated with increased GDH activity and higher insulin levels [132]. GDH activation via deacetylation by SIRT3 and GDH inactivation via ADP-ribosylation by SIRT4 (Figure 6) may be among mechanisms providing for targeted enzyme regulation under caloric restriction. Since caloric restriction increases SIRT3 expression and decreases SIRT4 expression, both GDH acetylation and ADP-ribosylation are decreased under these conditions, leading to GDH inactivation [132,135].

### 8.2. Brain GDH

Both a decrease and an up-regulation of GDH activity were shown to be damaging to brain. GDH deficiency is found in the brains of patients suffering from various neurodegenerative diseases [54,165]. GDH activity is significantly reduced in patients suffering from multisystemic atrophy, mainly affecting the cerebellum [166]. Significant changes in GDH activity are also observed in patients with olivopontocerebellar atrophy [167,168,169,170]. Further studies on the role of GDH in neurodegeneration showed that the loss of GDH function significantly increases glutamate levels, leading to brain damage due to excitotoxicity [171].

However, GDH up-regulation as a result of its gain-of-function mutations in such diseases as HI/HA syndrome considered above [172,173,174] is also damaging to brain. In addition to decreased glucose and increased ammonia levels, GDH up-regulation results in depletion of brain neurotransmitters glutamate and gamma-aminobutyric acid (GABA), for which glutamate is a precursor [173]. Inhibition of GABAergic signaling is known to contribute to dystonia, epilepsy and other neurological symptoms [173,175,176].

Recently, Plaitakis et al. have shown that a rare variant of the *GLUD2* gene, T1492G, resulting in a gain-of-function Ser445Ala substitution in hGDH2, is associated with development of a form of early onset Parkinson disease (PD) in a Caucasian populations [52]. This variant was shown to be even more resistant to GTP inhibition than is the wild-type hGDH2, but to possess a markedly increased sensitivity to inhibition by estrogens. The resulting inhibition of the overactive GDH by estrogens in female patients may result in protection from early development of PD [52]. This was further supported by the beneficial effects of estrogen administration in animal PD models [177,178].

Transgenic mice overexpressing GDH showed increased levels and release of brain glutamate, as well as a significant loss of neurons and reduction of dendritic spines and axon terminals [179]. Moreover, GDH overexpression resulted in up-regulation of several genes, including genes involved in oxidative stress, inflammation, cellular injuries as well as genes implicated in development of PD (e.g., α-synuclein gene) and Huntington disease [180].

Early Alzheimer disease (AD) was shown to lead to a significant increase in the levels of 3-nitrotyrosine residues in proteins, including nitrosylated GDH, whose activity is significantly decreased in some cases of early AD [181,182]. Nevertheless, other studies indicated that GDH protein level is increased in brain [183] and its activity is increased in plasma [184] of AD patients.

Elevated GDH levels were found in prefrontal cortex of schizophrenic patients, along with decreased levels of glutamine synthase [185,186]. According to the “glutamate hypothesis”, this may result in hypofunctioning of glutamatergic signaling leading to schizophrenia development. Known antipsychotic drugs were shown to be potent GDH inhibitors in vitro [187,188], with hGDH2 isoenzyme being markedly more sensitive to the drugs (IC50 = 26 μM for haloperidol and 31 μM for perphenazine), compared to hGDH1 (IC50 = 122 μM for haloperidol and 194 μM for perphenazine) [47]. The high sensitivity of hGDH2 toward these drugs suggests its involvement in the beneficial effects resulting from administration of antipsychotic drugs in schizophrenia patients, including modulation of the glutamatergic signaling by inhibition of hGDH2 [52].

It is worth noting that GDH in brain supplies 2-oxoglutarate for its oxidation, which occurs through the 2-oxoglutarate dehydrogenase complex (OGDHC), forming a supramolecular structure with GDH which in turn binds aspartate aminotransferase [189,190]. Since OGDHC dysfunction is also associated with neurodegeneration [191,192], the importance of GDH in the development of neurodegenerative processes may be mediated by impaired 2-oxoglutarate supply to OGDHC. In particular, overactivation of GDH leading to increased supply of 2-oxoglutarate to OGDHC may increase the 2-oxoglutarate-dependent production of reactive oxygen species by the complex [193,194].

### 8.3. GDH in Malignant Transformation

Glutamine dependence of tumor cells is linked to altered metabolism of glutamine and glutamate in malignancies [195,196]. After uptake by tumor cells, glutamine is converted to glutamate and ammonia by phosphate-activated glutaminase, whose activity has long been known to correlate with tumor growth [197,198,199]. Glutamate dehydrogenase reaction is also up-regulated in many tumors, which may increase the glutamate entrance into the Krebs cycle [200,201,202,203,204]. A high glutamate dehydrogenase activity has recently been shown to be a prognostic indicator of poor outcome in human colorectal cancers and gliomas [202,204].

One of the mechanisms for up-regulation of glutamate dehydrogenase activity in cancer cells is by inhibition of SIRT4 [122]. This increase in GDH activity correlates with the activation of mTORC1 [122]. GDH inhibition in glioma cells, coupled to SIRT4 activation, was shown to reduce glioma proliferation and inhibit tumor growth [204]. In glioblastoma cells, increased GDH expression enables cell survival upon glucose withdrawal by using glutamine as an alternative carbon source [200]. Suppression of GDH activity with siRNA to hGDH1, but not to hGDH2, decreases the viability of glucose-deprived glioblastoma cells [200]. A novel GDH inhibitor, the green tea polyphenol epigallocatechin-3-gallate, was also effective in several cancer models [200,205,206].

hGDH1 is involved in cancer redox homeostasis because 2-oxoglutarate and a subsequent metabolic intermediate of the Krebs cycle (fumarate) bind to and activate the ROS scavenging enzyme glutathione peroxidase 1 [201,204]. This is considered to be another factor contributing to glutamine dependence of tumor cells. The role of hGDH2 in cancer is much less investigated than is that of hGDH1, although this isoenzyme was shown to promote growth of glioma cells possessing a mutation in the isocitrate dehydrogenase 1 (*IDH1*) gene, in a manner that is not mimicked by overexpression of hGDH1 [207].

The importance of interplay between metabolism of glucose and glutamate in cancer cells has been shown upon inhibition of the 2-oxoglutarate dehydrogenase complex reaction at the pathways intercept [208]. Investigation of compensatory metabolic pathways and signaling functions of their intermediates may help to improve the efficacy of cancer therapies [200,207,208].

## 9. Conclusions

Results from extensive enzymological studies on GDH going back to the 1960s acquire new significance in view of an increasing body of structural data, both from resolved protein 3D structures and “omics” approaches. Combining old and new data increases our understanding of GDH and its metabolic role. Some non-canonical GDH features, which were earlier readily ascribed to artifacts, are undergoing a “re-discovery”, acknowledged and confirmed as biologically relevant many years after their initial presentation. Therefore, in addition to summarizing the well-known mainstream findings on GDH, this review is aimed at acknowledging also non-canonical data. We show how some of the “odd” localizations or multiple forms of GDH acquire significance, along with the development of a new scientific paradigm which takes into account the biological significance of not only widely known, but also moonlighting functions of well-characterized enzymes. Current progress in deciphering the structure–function relationship in mammalian GDH greatly increases resolution of our view on the enzyme and its metabolic roles, supported by multiple localizations, isoenzymes, and post-translational modifications. New studies characterizing these features continue to unravel the complex story of mammalian GDH regulation, which is still far from being complete. We expect that translation of this knowledge of GDH to the neurosciences and medical studies will be beneficial for solving the problems related to glutamate metabolism in health and disease.

## Figures and Tables

**Table 1:** Kinetic parameters of purified GDHs of animal origin in comparison to those of other higher eukaryotes. Michaelis constants *K_m_* and specific maximal activities, corresponding to *Vmax* per mg of protein (μmol/min per mg of protein) were measured at optimal pH values in the forward (Glu→2-OG) and backward (2-OG→Glu) GDH reactions. Glu—glutamate, 2-OG—2-oxoglutarate. GDHs were purified from total homogenate, except for GDH from pea stem [76] and turnip [77], which were purified from the mitochondrial fraction. Superscripts indicate the following variations in the assay conditions: ^a^—NAD(H)-dependent reaction; ^b^—NADP(H)-dependent reaction; *—1 mM ADP + 2.6 mM EDTA added; ^—0.3–0.4 mM CaCl_2_ added.

GDH Source	*K_m_*, mM							*V_max_* per mg of Protein	
	NAD^+^	NADP^+^	NADH	NADPH	Glu	2-OG	NH_4_^+^	Glu→2-OG	2-OG→Glu
**NAD(P)^+^-Dependent GDHs**									
**Animals**									
Human brain * [54]	*–*	*–*	0.08	0.05	17.7 ^a^	1.3 ^a^	*–*	*–*	160 ^a^
Rat brain * [27]	*–*	*–*	0.08	0.11	3.6 ^a^	1.4 ^a^	18.3 ^a^	*–*	168 ^a^
Bovine brain [55]	*–*	*–*	*–*	*–*	1 ^a^	*–*	*–*	*–*	40 ^a^
Bovine liver [78]	0.7	0.05	0.02	0.03	1.8 ^b^	0.7 ^b^	3.2 ^b^	1.92 ^b^	60 ^b^
Bovine liver [79,80]	0.18	0.004	0.02	0.02	0.7 ^a^; 0.9 ^b^	0.6 ^a^; 0.1 ^b^	74 ^a^; 38 ^b^	10 ^a^; 1.1 ^b^	67 ^a^; 38 ^b^
Chicken liver [81]	0.61	*–*	*–*	*–*	2.0 ^a^	*–*	*–*	5.9 ^a^	44 ^a^
Frog liver [82]	0.02	0.5	0.03	0.2	1.8 ^a^	5.0 ^a^	0.5 ^a^	1.0 ^a^	24 ^a^
Dogfish liver [83]	*–*	0.08	*–*	0.4	84 ^a^	4.5 ^a^	80 ^a^	3.0 ^a^; 0.5 ^b^	32 ^b^
**Plants**									
Duckweed [84]	0.18	*–*	0.02	*–*	2.5	1.5	29	*–*	57 ^a^
Duckweed [85]	0.46	*–*	0.11	0.13	12 ^b^	3.3 ^a^; 2.1 ^b^	27 ^a^; 1.5 ^b^	–	3.5 ^a^; 0.4 ^b^
Triticale roots [86]	0.53	0.48	–	0.06	18 ^a^; 19 ^b^	3.0 ^a^	4 ^a^; 0.1 ^b^	*–*	272 ^a^; 47 ^b^
**NAD^+^-Specific GDHs:**									
**Plants:**									
Pea seeds ^^^ [87]	0.23	*–*	0.03	*–*	9.3	2.3	52.6	90 ^a^; ~0 ^b^	530 ^a^; 8.5 ^b^
Pea roots [88]	0.65	*–*	0.86	*–*	7.3	3.3	72	8.6 ^a^; ~0 ^b^	49 ^a^; 7.5 ^b^
Pea stem [76]	0.24	*–*	0.09	*–*	12.5	5.6	68	*–*	27
Lupin nodules [89,90]	0.28	*–*	0.34	*–*	4.3	4.5	1010	25.3	1100
Turnip ^^^ [77]	0.25	*–*	0.09	–	28.6	2.0	44.4	–	450
**Fungi**									
*Neurospora crassa* [91]	0.33	*–*	0.55	*–*	5.5	4.6	17	*–*	590
*Candida utilis* [92]	1.08	*–*	*–*	*–*	20	*–*	*–*	47.7	414
**NADP^+^-Specific GDHs**									
**Fungi**									
*Agaricus bisporus* [93]	–	0.12	–	0.07	27	3.2	2.1	1.3	7.8
*Laccaria laccata* [94]	–	0.03	–	0.01	26	1	5	–	250
*Neurospora crassa* [91]	*–*	0.05	*–*	0.13	45	5.3	10	*–*	240
*Saccharomyces cerevisiae* [95]	*–*	0.07	*–*	0.09	10	1	110	18.9	227

**Table 2:** Michaelis constants for substrates (*K_m_*) determined for the mitochondrial and nuclear GDH from rat liver [26].

Substrate	Measurement Conditions	Mitochondrial GDH	Nuclear GDH
		*K_m_*, mM	*K_m_*, mM
Glutamate	50 mM K_3_PO_4_, pH 9.0, 0.05 mM NAD^+^	4.35	0.91
2-oxoglutarate	50 mM K_3_PO_4_, pH 7.6, 0.05 mM NADH, 50 mM NH_4_Cl	0.45	0.13
NH_4_^+^ (NH_4_Cl)	50 mM K_3_PO_4_, pH 7.6, 0.05 mM NADH, 1.25 mM 2-oxoglutarate	30.0	11.0
NAD^+^	50 mM K_3_PO_4_, pH 9.0, 25 mM glutamate	0.02	0.06
NADH	50 mM K_3_PO_4_, pH 7.6, 50 mM NH_4_Cl, 1.25 mM 2-oxoglutarate	0.02	0.02

**Table 3:** Comparison of kinetic parameters of the overexpressed human GDH isoenzymes, either purified [50] or in cell extracts [101], and the multiple forms of GDH from bovine brain [103]. TEA—triethanolamine, NH_4_OAc—ammonium acetate, 2-OG—2-oxoglutarate, Glu—glutamate. The ADP concentrations (in bold) are important to note when comparing the differences in ***K_m_***, exhibited by the GDH isoforms.

Varied Substrate	Assay Conditions, Purified Human GDH [50]	*K_m_*, mM		Assay Conditions, Human GDH in Cell Extracts [101]	*K_m_*, mM		Assay Conditions, Purified GDH from Bovine Brain [103]	*K_m_*, mM	
		hGDH1	hGDH2		hGDH1	hGDH2		bGDH1	bGDH2
Glu	50 mM TEA, pH 8.0, 1.4 mM NADP^+^, 2.6 mM EDTA, 1 mM ADP	12.4 ± 0.7	10.7 ± 0.8	50 mM TEA, pH 8.0, 1.4 mM NADP^+^, 2.6 mM EDTA, 0.1 mM ADP	7.6 ± 1.0	2.4 ± 0.4	50 mM Tris/HCl, pH 9.5, 1.4 mM NADP^+^, 2.6 mM EDTA, 1 mM ADP	8.3	3.4
2-OG	50 mM TEA, pH 8.0, 0.1 mM NADPH, 0.1 M NH_4_OAc, 2.6 mM EDTA, 1 mM ADP	2.0 ± 0.2	2.1 ± 0.3	50 mM TEA, pH 8.0, 0.15 mM NADPH, 0.1 M NH_4_OAc, 2.6 mM EDTA, 0.25 mM ADP	0.9 ± 0.1	1.5 ± 0.02	50 mM TEA, pH 8.0, 0.1 mM NADPH, 0.1 M NH_4_OAc, 2.6 mM EDTA, 1 mM ADP	1.3	2.2
NH_4_OAc	50 mM TEA, pH 8.0, 0.1 mM NADPH, 8 mM 2-OG, 2.6 mM EDTA, 1 mM ADP	13.4 ± 0.7	17.1 ± 2.0				50 mM TEA, pH 8.0, 0.1 mM NADPH, 10 mM 2-OG, 2.6 mM EDTA, 1 mM ADP	15.4	20.0
NAD^+^							50 mM Tris/HCl, pH 9.5, 25 mM Glu, 2.6 mM EDTA, 1 mM ADP	0.8	0.9
NADP^+^								1.2	1.3
NADH							50 mM TEA, pH 8.0, 0.1 mM NADH, 10 mM 2-OG, 2.6 mM EDTA, 1 mM ADP	0.12	0.07
NADPH								0.1	0.1
